# New progress in diagnosis and treatment of pulmonary arterial hypertension

**DOI:** 10.1186/s13019-022-01947-y

**Published:** 2022-08-29

**Authors:** Zai-qiang Zhang, Sheng-kui Zhu, Man Wang, Xin-an Wang, Xiao-hong Tong, Jian-qiao Wan, Jia-wang Ding

**Affiliations:** 1 Department of Cardiology, The First College of Clinical Medical Sciences, China Three Gorges University, 183 Yiling Road, Yichang, 443000 Hubei People’s Republic of China; 2 Institute of Cardiovascular Diseases, China Three Gorges University, Yichang, 443000 Hubei Province People’s Republic of China

**Keywords:** Pulmonary arterial hypertension (PAH), Pathophysiology, Biomarker, Genetic

## Abstract

Pulmonary arterial hypertension (PAH) is a progressive disease. Although great progress has been made in its diagnosis and treatment in recent years, its mortality rate is still very significant. The pathophysiology and pathogenesis of PAH are complex and involve endothelial dysfunction, chronic inflammation, smooth muscle cell proliferation, pulmonary arteriole occlusion, antiapoptosis and pulmonary vascular remodeling. These factors will accelerate the progression of the disease, leading to poor prognosis. Therefore, accurate etiological diagnosis, treatment and prognosis judgment are particularly important. Here, we systematically review the pathophysiology, diagnosis, genetics, prognosis and treatment of PAH.

## Introduction

Pulmonary arterial hypertension (PAH) is a progressive disease of the pulmonary vasculature characterized by pulmonary vasoconstriction and progressive occlusion of the distal pulmonary artery (PA), which can lead to elevated pulmonary artery pressure, right ventricular (RV) failure and death [1]. Its pathogenesis is complex and involves endothelial dysfunction, chronic inflammation, smooth muscle cell proliferation, pulmonary arteriole occlusion, apoptosis resistance and pulmonary vascular remodeling. PAH arises as a consequence of many different etiologies, including external factors (hypoxia, tobacco, dust, and other physical and chemical biological factors), internal factors (genetics, development, structure, and disease) and interactive factors (microecology, infection, immunity, and drugs). PAH is mainly characterized by remodeling of the pulmonary arteries, in which the smooth muscle layer of the vessel wall thickens and abnormal neointima cells accumulate beneath the endothelial layer. This leads to pulmonary vascular remodeling, narrowing of the pulmonary vascular bed and a progressive increase in pulmonary artery total resistance, which eventually occludes the vessel lumen, increases pulmonary vascular resistance and leads to right heart failure [2]. No effective treatment has been discovered yet. In this paper, we summarized the research related to PAH in recent years and reviewed the pathophysiology, diagnosis, genetics, prognosis and therapy for PAH.

## Classification and pathophysiology of PAH

PAH is a persistent and progressive disease that often leads to premature death. The most common etiology is left heart or lung disease. According to epidemiological data on PAH, the incidence of PAH is up to 7.6 cases/million adults, and the prevalence is up to 26–100/million adults worldwide [3]. The morbidity and mortality of PAH are significantly high. Early diagnosis and treatment are essential. According to the latest clinical classification of PAH by the European Society of Cardiology (ESC) and the European Respiratory Society (ERS) [4], PAH can be categorized into 5 groups: (1) pulmonary arterial hypertension (PAH), which comprises diverse diseases that result in similar pathological changes within the pulmonary vasculature; (2) PAH caused by left heart disease, such as systolic or diastolic heart failure and left-sided valvular diseases; (3) PAH caused by pulmonary disease and/or hypoxia; (4) PAH caused by chronic thromboembolic embolism (CTE) and other pulmonary obstructive processes; and (5) PAH associated with unclear or multifactorial mechanisms.

The pathogenesis of PAH is intricate and mainly involves the distal pulmonary arterioles, which are characterized by the proliferation of the pulmonary artery intima with inflammatory reaction, endothelial stroma, thickening of the pulmonary artery intima and formation of plexiform lesions due to the hypertrophy and continuous contraction of media, adventitial fibrosis, matrix remodeling, and inflammatory infiltration around pulmonary arterioles [5]. The distal end of the lesion dilates, and a thrombus forms in situ, resulting in progressive stenosis and occlusion of the pulmonary artery lumen. Clinical evidence indeed indicates that chronic persistent inflammation is present in PAH and contributes to disease progression [6]. It has also been shown that a wide array of inflammatory markers are increased in the serum of PAH patients and correlate with disease severity or patient survival. Previous studies have found that the proinflammatory cytokines IL-1a, IL-1b, IL-6, TNF-a and IL-13 were associated with an increased risk of death in PAH patients [6]. Soon et al. also found that the serum levels of IL-2, IL-4, IL-8, IL-10 and IL-12p70 were increased in patients with idiopathic and familial PAH and that the circulating levels of IL-2, IL-6, IL-8, IL-10 and IL-12p70 had a significant effect on survival[7]. Numerous studies have also demonstrated that perivascular inflammatory infiltrates in PAH lungs are constituted by macrophages and monocytes, T and B lymphocytes, cytotoxic and helper T cells, natural killer cells, dendritic cells (DCs), and mast cells [8–10]. Pulmonary artery smooth muscle cells (PASMCs) and pulmonary artery endothelial cells (AECs) play an important role in the pathophysiological process of PAH. The failure of endothelial cell apoptosis might result in apoptosis-resistant and hyperproliferative vascular endothelial cells and smooth muscle cells. Jurasz et al. also found decreased apoptosis based on caspase-3 activity in endothelial cells isolated from the pulmonary arteries of patients with idiopathic PAH, and the antiapoptotic protein BCL-2 was overexpressed in the lungs of patients with irreversible PAH [11]. The pathophysiological mechanism of PAH is complex and is the result of many factors. Further research on the mechanism is needed to provide a new method for the development of treatment.

## Biomarkers of PAH

Once PAH is diagnosed, its prognosis is often poor, as there is still a lack of drugs that can effectively prevent the progression of PAH and improve its prognosis. It is very important to accurately judge the severity of the disease, prognosis and response to treatment. Therefore, it is of great value to find markers that can reflect the condition and prognosis. At present, a variety of biomarkers have been confirmed to be of great significance in the diagnosis, treatment, efficacy evaluation and prognosis of PAH. Previous studies have found many markers that can predict the prognosis of PAH, including clinical signs of RV failure, such as right-sided volume overload, the rapidity of the progression of symptoms and syncope, functional class, 6-min walk distance (6MWD), cardiovascular stress testing, biomarkers indicative of myocardial stress, and hemodynamic variables (right atrial pressure, cardiac index, and central venous saturation) [4].

BNP/NT-proBNP are markers of myocardial stress and dysfunction. Although BNP has no obvious advantage over NT-proBNP, BNP has a strong correlation with the hemodynamic effects of PAH in addition to being less affected by kidney function, while NT-proBNP seems to be a better predictor of prognosis. A study found that a higher level of BNP and NT-proBNP was found in patients with PAH secondary to congenital heart disease (CHD) than in healthy controls [11]. Recent data showed that a baseline NT-proBNP level of 340 ng/L strongly predicted the 5-year survival rate of PAH patients [12]. The NT-proBNP category has a high predictive value for long-term outcomes by using the NT-proBNP baseline quartile and ESC/ERS guideline threshold in the GRIPHON post hoc analysis [13]. These analyses also indicate that the NT-proBNP category can be used to predict the long-term treatment response during follow-up. The ESC/ERS guidelines recommend the use of NT-proBNP as a part of a multiparameter prognostic assessment for prognosis and as a treatment goal for PAH patients and provide thresholds to define low (< 300 ng/L), moderate (300–1400 ng/L) and high-risk (> 1400 ng/L) NT-proBNP levels [4]. All these results indicate that BNP/NT-proBNP is highly predictive of long-term outcomes in PAH patients at any time of examination, during treatment and in follow-up.

Survivin is the smallest member of the family of apoptosis protein inhibitors [14]. The established molecular characteristics of survivin include inhibition of apoptosis, promotion of cell proliferation and promotion of tumor angiogenesis [15, 16]. Survivin is virtually undetectable in normal adult differentiated tissues but has been found to be highly expressed in a variety of malignant tumors [17]. Overexpression of survivin has been shown to strongly inhibit cell death in many cells. Li et al. found that irreversible PAH rats showed significant obstructive lesions caused by intimal formation, which was associated with a decrease in apoptosis and an increase in survivin expression [18]. However, reversible PAH rats were characterized by medial hypertrophy resulting in mild occlusion, with increased apoptosis and unchanged survivin expression [18]. In addition, compared to both reversible PAH and control rats, the serum survivin level of irreversible PAH rats was significantly increased and was positively correlated with the expression of survivin in the lung [18]. Survivin plays an important role in predicting the prognosis of PAH in patients with congenital heart disease, especially in evaluating the reversibility of PAH in patients with this condition. The study found that the preoperative serum survivin level of patients with irreversible CHD-PAH was significantly higher than that of patients with reversible CHD-PAH. There was also a significant correlation between the serum survivin level and BNP, the preoperative pulmonary vascular resistance index and postoperative mean PAH [19]. This finding suggests that the increase in survivin levels is a characteristic of irreversible PAH, and serum survivin is a selective biomarker reflecting the prognosis of CHD-PAH patients.

Tet methylcytosine dioxygenase-2 (TET2) is a key enzyme of DNA demethylation that plays an important role in cardiovascular disease and hematological disease and is related to clonal hematopoiesis, inflammation and adverse vascular remodeling [20, 21]. Potus et al. found that Tet2 knockout mice spontaneously developed PAH, with adverse pulmonary vascular remodeling and an inflammatory response, accompanied by increased levels of cytokines, including IL-1β [22]. Long-term targeting of IL-1βblocking of the antibody could induce the regression of PAH [22]. This experimental observation suggests that total or partial loss of TET2 function can induce vascular remodeling secondary to increased inflammation in animal models. In an independent cohort, TET2 expression was decreased in more than 86% of associated pulmonary arterial hypertension (APAH) and idiopathic pulmonary arterial hypertension (IPAH) patients [23]. This observation indicates that PH is related to TET2 mutations in both humans and mice. All these results indicate that TET2 may be used as a biomarker of PAH.

Cartilage intermediate layer protein (CILP1), an antagonist of transforming growth factor β (TGF-β), is an extracellular matrix (ECM) protein that is involved in profibrotic signaling in the myocardium [24]. In an animal model of left ventricular pressure overload and left ventricular myocardial infarction, the expression of CILP1 mRNA was upregulated, and the level of CILP1 protein was significantly increased in patients with aortic stenosis or myocardial infarction [24, 25]. Diastolic stiffness is characterized by increased fibrosis and intrinsic stiffening in the right ventricular cardiomyocyte sarcomeres, which is closely related to the severity of pulmonary arterial disease [26]. It was found that the protein level of CILP1 was significantly increased in mice with PAH [27]. In PAH patients with maladaptive RV function, the CILP1 concentration was higher than that of pH patients with adaptive RV function, and CILP1 had good predictive ability in ROC analysis [28]. Therefore, CILP1 may be a novel biomarker of RV and LV pathological remodeling in patients with PAH, which is associated with worse prognosis.

The etiology of PAH is complex, and the treatment effect is poor. At present, several studies have also found that troponin I, TIMP-1, TIMP-2 and IGFBP-2 are associated with severe diagnosis, prognosis, and treatment effect evaluation of PAH patients [29, 30]. Therefore, we need more research to identify new biomarkers to further improve the accuracy of diagnostic work-up.

## Genetic characteristics of PAH

Many studies have found that PAH is associated with genetic variation. Epidemiological studies have found that approximately 6–10% of PAH patients have familial segregation, among which PAH has a monogenic autosomal dominant inheritance with incomplete penetrance, ranging from 14% in males to 42% in females [31]. The incidence of PAH in women was significantly higher than that in men. At present, it has been found that a variety of gene changes are related to the occurrence of PAH. Bone morphogenetic protein receptor 2 (BMPR2) mutations are critical risk factors for hereditary pulmonary arterial hypertension (hPAH), with approximately 20% of carriers developing the disease [32]. In 2000, BMPR2 gene mutations were first identified in several families with HPAH [33, 34]. Subsequent studies have confirmed that BMPR2 mutation is the most common genetic cause of PAH, accounting for approximately 80% of hereditary PAHs (HPAHs) and 20% of idiopathic PAHs (IPAHs) [35]. Wen et al. found that in BMPR2-deficient rats, it can induce pulmonary inflammation and severe pulmonary hypertension by mediating the overexpression of inflammatory factors [36]. In population statistics, the penetrance of BMPR2 mutation in women (42%) is higher than that in men (14%) [31]. Current guidelines classify sporadic PAH cases with BMPR2 mutations as HPAH. The data indicate that 25% of PAH-specific mutations in BMPR2 were missense mutations leading to amino acid substitutions in both the idiopathic and familial forms of PAH. In contrast, it was predicted that the vast majority of mutations would result in premature protein truncation due to nonsense mutations (27%), frameshift mutations caused by small nucleotide insertions or deletions (23%), gene rearrangements (14%) or splice site mutations (10%) [33, 34, 37, 38].

Recent studies have also found that CAV1 and KCNK3 gene mutations are associated with PAH [39, 40]. CAV1 encodes a membrane protein located in the caveolae, which are rich in cell surface receptor proteins, including TGF-b family members [39]. Austin et al. confirmed that CAV1 mutation was associated with the severity of PAH [39]. Studies with CAV1 knockout mice also found that these mice suffered from pulmonary hypertension with medial thickening, muscularization of distal pulmonary vessels and loss of total pulmonary artery surface area, as well as thickened alveolar septa, hypercellularity and an increase in extracellular fibrillar deposits [41, 42]. The expression of CAV1 was also decreased in monocrotaline-induced pulmonary hypertension [43]. NOS3 gene deletion studies have shown that the occurrence of pulmonary vascular disease in CAV1-deficient mice is due to the hyperactivity of eNOS and subsequent tyrosine nitration-dependent impairment of protein kinase G (PKG) activity, which is also supported by data obtained from human IPAH [44]. KCNK3 encodes a potassium channel, and its mutation may cause PAH through a mechanism that is different from TGF-b signaling. Ma et al. reported kCNK3 channel gene mutation in patients with PAH, which is a cause of HPAH [40]. Pulmonary veno-occlusive disease (PVOD) and pulmonary capillary hemangiomatosis (PCH) belong to the PAH subgroup and are characterized by obvious venous or capillary involvement. Recently, EIF2AK4 mutations have been found in familial and sporadic PCH and PVOD patients [45, 46], in which severe PAH occurs due to histopathological abnormalities at the level of pulmonary capillaries and small venules, respectively [47]. It is usually associated with more severe hemodynamic derangements and more rapid disease progression [47]. Two recent studies have shown that a small proportion of patients clinically diagnosed with PAH carry EIF2AK4 mutations [48, 49]. Another study further confirmed the pathogenic role of EIF2AK4 mutations in patients clinically diagnosed with PAH [49].

In addition, Wang et al. recently discovered prostaglandin synthase (PTGIS), a novel disease gene that codes for rare idiopathic pulmonary hypertension (IPAH), which can explain 6.1% of IPAH patients [50]. The study also found that patients with rare mutations of PTGIS are more sensitive to prostacyclins, thereby providing the rationale for targeting the prostacyclin pathway as part of tailored therapies for managing patients with PTGIS variants [50]. Shinya et al. also found that the TNFRSF13B p.Gly76Ser variant may be involved in the development of PAH via aberrant inflammation in pulmonary vessels [51]. With the deepening of genetic research on PAH, an increasing number of related pathogenic genes have been found. These data emphasize the importance of accurate gene diagnosis for the effective management and treatment of PAH patients.

## Specific medications of PAH

**Fig. 1:**
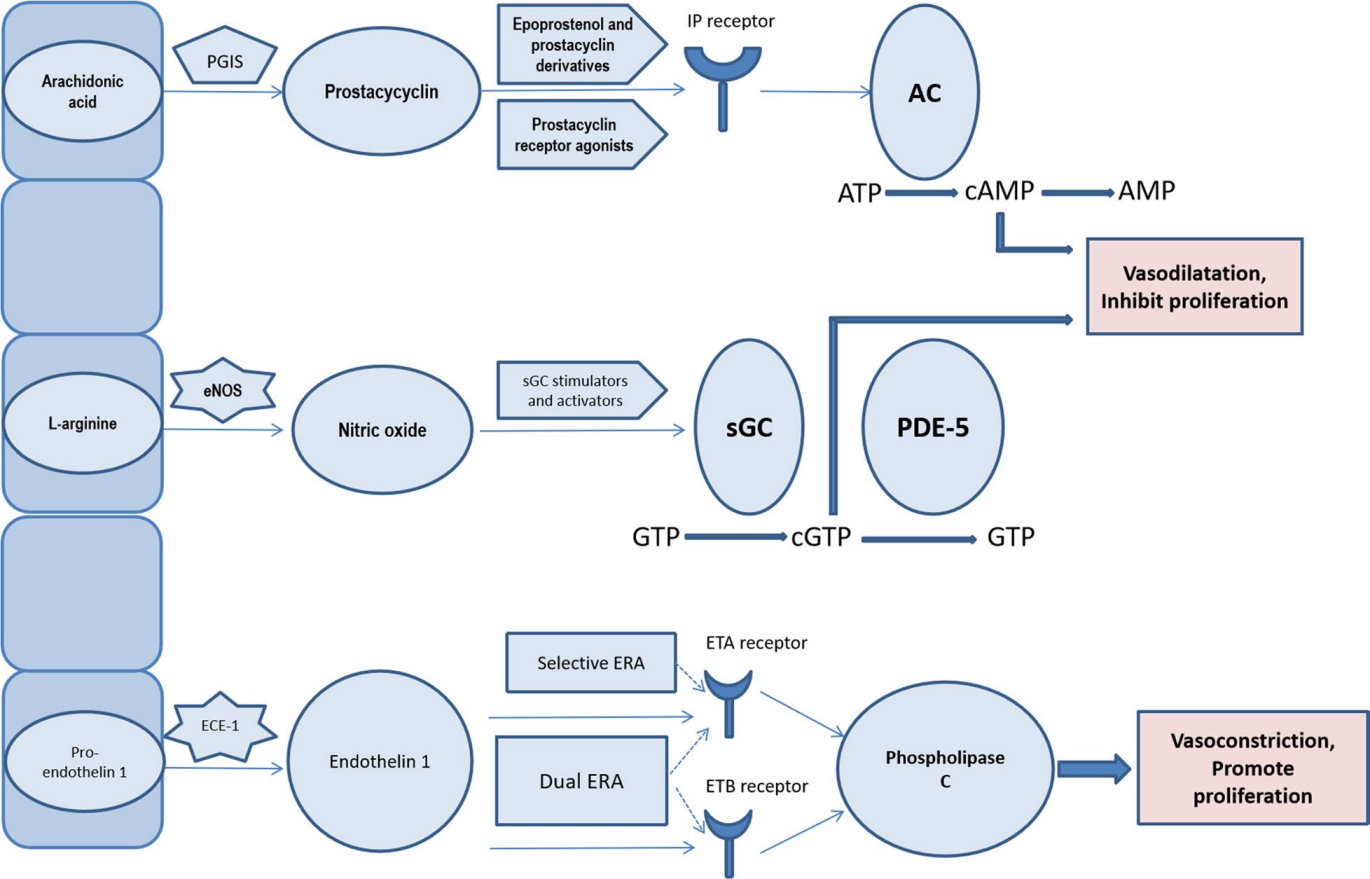
Current and emerging targets and therapies for pulmonary arterial hypertension. Arrows represent receptor stimulation, whereas dashed arrow show receptor blockade. Abbreviations: PGIS, prostaglandin I synthase; IP, prostaglandin I2; AC, adenylate cyclase; cAMP, cyclic AMP; cGMP, cyclic GMP; eNOS, endothelial nitric oxide synthase; sGC, soluble guanylate cyclase; PDE-5, phosphodiesterase type 5; ECE-1, endothelin converting enzyme 1; ETA, endothelin receptor type A; ETB, endothelin receptor type B; ERA, endothelin receptor antagonists

At present, the available drugs for the treatment of PAH remain reliant on differential action in 3 well-established pathways. These pathways are the antagonism of the prostacyclin and nitric oxide pathways and the antagonism of the endothelin pathway (Fig. 1). Although there is no specific drug for the treatment of PAH, modern PAH-specific treatment can improve exercise tolerance and quality of life and, in some cases, improve the time to clinical worsening and reduce mortality.

### Endothelin receptor antagonists (ERAs)

Endothelin plays an important role in the pathogenesis of PAH. Endothelin-1 can competitively inhibit the binding of type A and type B endothelin receptors in pulmonary vascular smooth muscle and endothelial cells, cause vasoconstriction, promote mitosis, and participate in the occurrence and development of PAH [52]. ERAs can treat PAH by inhibiting endothelin. At present, the main ERAs are bosentan, ambrisentan and macitentan, which selectively block the effects of ET-1 on several receptors. Bosentan, which is a dual antagonist of endothelin receptors A and B, was the first orally available medication for the treatment of PAH [53]. It has been shown to improve exercise capacity, clinical deterioration time and various hemodynamic measures in patients with WHO FC II-IV symptoms compared with placebo [54]. Bosentan has certain teratogenicity and hepatotoxicity, necessitating monthly assessment of liver function. Alisentan is a highly selective endothelin A receptor antagonist [55]. Studies have shown that alisentan can significantly improve exercise tolerance and hemodynamic parameters in patients with PAH [56]. It has less potential for hepatotoxicity than bosentan; however, it is associated with more peripheral edema [56]. Maxitengtan is a new generation of dual ERAs with better tissue penetration and receptor affinity. A number of studies have shown that maxitengtan can significantly reduce the mortality or hospitalization rate of patients with PAH and improve 6MWD, cardiac function, quality of life, hemodynamic parameters and NT–proBNP[57–59].

### Prostacyclin analogues

Prostacyclins act on the prostacyclin pathway to increase cAMP, promote vasodilation and inhibit both platelet aggregation and smooth muscle cell proliferation [60]. Studies have shown that the expression of prostacyclin synthetase in the pulmonary artery of PAH patients is decreased, and the metabolic level in urine is also decreased. Synthetic prostacyclin analogs can be used for the treatment of PAH [61]. Epoprostenol has been reported to have antiproliferative, antiplatelet and anti-inflammatory effects [62]. Epoprostenol improves symptoms, 6MWD and hemodynamics in patients with PAH. The extent of the side effects of epoprostenol may be patient- and dose-dependent [4]. As time passes, the drug dose needs to be increased to reach an effective level. Sudden withdrawal can lead to fatal rebound pulmonary hypertension. Traprost is a synthetic prostanoid molecule that has the advantage of a longer half-life than epoprostenol and room temperature stability [63]. Data from a large multicenter randomized controlled trial show that subcutaneous injection of traprost improves hemodynamics, 6MWD, and subjective symptoms in patients with PAH [64]. Selexipag is a unique prostacyclin receptor agonist that can be used as an oral drug with active metabolites. Selexipag reduced the rate of a composite end point of death or a complication related to PAH compared with placebo [65]. Adverse reactions to these drugs include jaw pain, nausea/vomiting and musculoskeletal pain. Additional distinctive adverse effects included thrombocytopenia (epoprostenol), gram-negative bacteremia (IV treprostinil), and dose- and treatment-limited infusion site pain/erythema (treprostinil) [66].

### PDE-5 inhibitors

NO is an important vasodilator that can achieve vasodilator effects by maintaining the concentration of cGMP in vascular smooth muscle cells. Pulmonary vessels contain a lot of PDE5, which is a cGMP-degrading enzyme. PDE-5 inhibitors target the NO pathway by preventing the breakdown of cGMP [60]. In addition, PDE5 inhibitors have antiproliferative effects. The main therapeutic effect of sildenafil and tadalafil is to facilitate vasodilation of vascular smooth muscle via the secondary messenger cGMP. Compared to placebo in patients with predominantly WHO FC II and III symptoms, sildenafil and tadalafil have been shown to improve exercise capacity [67]. Sildenafil has also been shown to improve hemodynamics and WHO FC in patients receiving initial treatment, while tadalafil is associated with improvement in the time to clinical worsening (TTCW) in patients receiving initial treatment [67–69]. The main adverse effects of these drugs are related to vasodilation, including headache, flushing, indigestion, myalgia, epistaxis, and visual and auditory alterations.

### Soluble guanylate cyclase stimulators (sGCS)

Soluble guanylate cyclase stimulators (sGCS) can also directly stimulate sGC independently to increase cGMP by acting together with endogenous NO to target the NO pathway. As a new sGC agonist, riociguat has a unique dual activation mechanism of sGC [70]. Its effect does not depend on the level of NO in vivo. It can increase the level of cGMP in plasma alone or in combination with NO, causing vasodilation and anti-remodeling [71]. Riociguat has been shown to improve 6MWD, WHO FC, NT-proBNP, and PVR and to delay TTCW compared to placebo in patients with WHO FC II to III symptoms [72]. Adverse effects of riocguat include headache, dizziness, hypotension, syncope and indigestion. Due to its teratogenic effects, female patients need defined contraception requirements.

### Calcium channel blockers (CCBs)

Patients with a positive acute vascular reaction test should be given enough CCBs. CCBs can significantly improve short-term hemodynamics in PAH patients with positive acute vasodilator testing [73]. Current guidelines suggest that other classes of PAH-specific therapies should be added to patients who do not achieve functional class I or II status and near normalization of hemodynamics on CCB therapy [74].

### Other PAH-specific medications

With the deepening of research on PAH, an increasing number of new specific medications have been uncovered. Recent studies have found that the Rho kinase inhibitor fasudil improves the acute hemodynamics of congenital heart disease with severe pulmonary hypertension and that the high dose was more effective than the low dose in treating congenital heart disease with severe PAH [75]. In addition, studies have found that silibinin, a CXCR4 inhibitor, can ameliorate PAH and reduce pulmonary arterial pressure phases, but it is an ineffective treatment in the late stages of the disease [76]. In addition, silibinin may delay pulmonary artery occlusion and pulmonary vascular remodeling by inhibiting the CXCR4/SDF-1 axis [76]. The discovery of these new specific medications has brought new hope to patients with PAH, but the efficacy, durability, safety, and long-term clinical impact of these PAH-specific medications for PAH patients need to be further evaluated.

## Surgical treatment of PAH

At present, the treatment of chronic thromboembolic pulmonary hypertension (CTEPH) mainly includes drug therapy, surgical pulmonary endarterectomy (PEA), pulmonary artery balloon dilatation and lung transplantation. In recent years, with the rapid development of surgical and interventional techniques, an increasing number of patients with PAH have been treated by surgery. Since the late 1990s, atrial septostomy has been used as an alternative palliation to transplantation in patients with severe and progressive PAH[77]. In most PAH patients, end-stage disease is characterized by right ventricular failure with falling systolic pressures and rising end diastolic and right atrial pressures. Therefore, atrial septostomy can "decompress" the failed right ventricle by allowing right to left atrial shunt to improve left ventricular preload and in turn augment cardiac output [78]. In 2004, a group from Paris, France, launched a new interventional treatment program for children with severe PAH [79]. This group treated severe PAH by creating a surgical anastomosis between the left pulmonary artery and the descending aorta. By creating an unlimited shunt between the pulmonary artery system and the systemic artery system, the afterload of the right ventricle will be reduced from the suprasystemic to systemic levels [79]. In addition, for CTEPH, a multicentre study from the United States evaluated 750 patients diagnosed within 6 months and described the differences in baseline characteristics and 1-year outcomes between patients who did and patients who did not undergo surgery [80]. It was found that patients who underwent PEA had the most favourable outcomes, with a low 30-day mortality rate of 3.9% and 82.9% of patients showing a reduction to NYHA functional class 1 or 2 at 1 year [80]. In addition, for patients with residual PAH after pulmonary endarterectomy, pulmonary artery denervation (PADN) in patients with CTEPH after PEA was safe and effective and significantly reduced pulmonary vascular resistance (PVR) and pulmonary artery pressure (PAP) during the 12-month follow-up while improving the 6MWT distance and reducing the need for hospitalization [81]. The findings from a meta-analysis published in Clinical Cardiology showed that balloon pulmonary angioplasty could improve the pulmonary haemodynamics and exercise tolerance of some inoperable patients with CTEPH compared to riociguat therapy [82].

## Conclusions

PAH is a complex disease with high mortality and disability. Over the past several decades, there has been significant progress in the understanding of the pathophysiology, genetics, diagnosis and treatment of PAH. However, there is no effective treatment for PAH. Therefore, the severity and risk of PAH patients should be evaluated to develop effective treatment strategies. In addition, patients also need regular structured follow-up, accurate assessment of the patient’s condition, and timely detection of disease changes to adjust the treatment strategy.

## Data Availability

Not applicable.
